# Malingering—No. 2

**Published:** 1862-11

**Authors:** Tom. O. Edwards

**Affiliations:** Chicago


### MALINGERING.—No. II.

By TOM. O. EDWARDS, M. D., Chicago.

You were kind enough to print my article on Malingering, and I fulfill my promise to continue the subject. In that communication I referred to increased and diminished size of parts, to the production of tumors by inflating the subcutaneous tissue with air, the similation of ascites hydrocephalus, dropsies of joints, malformation of joints, curvatures of the spine, wounds, ulcers, superficial inflammations, spasmodic affections, as epilepsy and hysteria and paralytic affections, and endeavored briefly to point out the diagnostic signs between the real and feigned diseases mentioned. There came under the classification of diseases obvious to the senses another class of diseases not obvious to the senses, but dependant upon the description of the impostor. We may divide this class into diseases of exalted or reduced sensibility, of pain, or absence of sensation.

The obscurity which ever has and ever will enshroud the nervous system, render the efforts of the physiologist in his enquiries of the cause of exalted sensibility in pain, extremely obscure and unsatisfactory. Increased sensibility in a part may exist and no visible sign of its presence, hence this symptom is more frequently assumed than any other from the great success and unanimity with which it is practiced. There are many external pains, such as tic doloreux, the causes of which are obscure, and which leave no visible sign of their existance, and yet no disease is more formidable in suffering or less amenable to treatment. Men have committed suicide rather than endure the torture of a disease, which externally gave no evidence of its presence. Upon the patient's statements we are compelled to rely for information in relation to the seat and violence of such affections, and we cannot be too careful in giving opinions in a disease the only element or phenomenon of which is pain, as gross frauds may be the result of our diagnosis, or patients in real disease may be regarded as feigning. The nature of the pain, the presence or absence of the symptoms of disease with which it is ordinarily associated, the general character and appearance of the patient, and the consistent account given of the case are important items for observation. We should guard opinions in examination of diseases in which pain alone is the cause of complaint, and should rely on time and accident to expose the fraud. Internal pains are mostly assumed, as those on the exterior are regarded as trivial, and a train of phenomena, such as heat, redness or unnatural color, are not unusually attendants on the real suffering. Gout and Rheumatism are favorites with Army Malingerers. Neither of these diseases can exist in severity without inducing changes in their locality. Heat, redness, tumefaction or retraction of the parts are characteristic of these diseases. True, there may be slight cause of complaint, and from peculiarity of organization or fretfulness of temper the case may be magnified. We have also pains, severe and long-continued, from scurvy and syphilis, the seat of which cannot be ascertained. The Malingerer will seldom submit to severe agents in these assumed external diseases. He will most commonly ask if nothing milder could be used, and if his request be acquiesced in, he will be better or worse until he is assured severe treatment will be instituted and then he is suddenly cured.

Internal pains selected by one feigning illness will, if properly managed, lead to detection. Thus a patient complains of constant agonizing pain in the head; he has no vertigo, no loss of sleep, fever or delirium; he says he has violent, deep-seated pain in the chest, and neither cough, difficult respiration nor fever be present; pain in the stomach and bowels, and neither impaired digestion nor loss of appetite be present; pain in the kidneys, and there is no retraction of the testicle nor diminution or alteration in the urine. Should any or all these necessary attendants upon these diseases be absent, and only pain be complained of, we have grounds of suspicion which it would be well not to disregard. If these pains are said to be constant and intensely violent, and yet the patient sleeps well and enjoys his food, there is room for doubt. We should avoid the exposure of our suspicions and be careful in our prescriptions. If we desire to place him under the influence of anodynes, they should be administered in food, as the sagacity of the patient may detect the purpose and he will measurably thwart our purpose.

The most astute minds have been imposed upon by the assumption of pain, and long, protracted, fatal suffering has been declared feigned, and the patient deprived of sympathy and kindness. Fodere relates a case in which he mistook real pain for feigned. "I refused (he says) for fifteen years, a certificate of exemption to a young soldier, who complained of violent pains, sometimes in one limb, and sometimes in another, and occasionally in the thorax and pericranium, without any external sign to indicate its existence. He died in the hospital from the effects of the malady—which he always insisted was a species of rheumatism. I examined the body after the death—viewed all the former seats of disease—but discovered nothing, either in the membranes, muscles, nerves, or viscera, and was hence led to believe that life was destroyed solely by the repetition and duration of these pains." Bitter was his mortification and regret that he had persisted so long in opposing the wishes and depriving the soldier of his discharge, and in the leniency of the remembrance of this case, the following one was presented to him. An artillerist from the Hart de Bonce, was brought to the hospital with a violent pain in his left leg, which was attributed to sleeping on the damp ground. During the space of eight months, a variety of antimonial preparations, together with mercurials and tonics, when indicated, were administered, with local remedies, but without any relief. The leg, from the repeated use of epispastics and cauteries, became thin, and rather shorter than the other, while from the low diet ordered there was a general paleness and lankness of the system. Under these circumstances, Fœdere could not refuse him a certificate as a real invalid. With the aid of his crutches he dragged himself to Versailles, where he obtained the promise of a discharge. He was ordered to return to the fort to await its arrival; but on his way thither, being overjoyed, he was met by his commander, walking without his crutches. On being put into prison, he confessed the fraud. It will require close observation and an intimate acquaintance with real disease to detect those of which pain is the most prominent symptom, as no words can adequately describe the difference. A general set of phenomena must be carefully sought and traced to their origin, such as gesture, expression of countenance, posture, and uniformity of symptoms.

Diseases attended by diminished sensibility are frequently assumed, and sometimes successfully. The most common is Amaurosis, and so successfully can this be assumed, that no possible means, other than careful watching, can detect the fraud. The pupil is dilated; the eye is learned to roll, and a feigned inability to fix the sight upon an object, are the symptoms. These are produced by the use of agents that act upon the nerves of the retina, and produce dilatation. Hyoscyamus, Belladona, etc., are locally employed in cases of this disease; when recent we may by carefully examining the patient be enabled, by the absence of all gastric derangements and febrile phenomena, to be put upon our guard and by putting a strict watch upon the actions of the patient, time will develope the rest. Fodere recommends the placing of the patient on a precipice, and ordering him forward. If there be real blindness, this procedure would be cruel and fatal, and was exhibited in a case of a recruit who was suspected of assuming blindness, being put on the bank of a river and ordered forward. He went forward and was rescued, but afterward confessed the fraud. Myopia, Amblyopia, Nyctalopia, Hemaolopia are all diseases found among Malingerers. We detect the Myopist feigner by putting on him glasses worn by persons known to be thus afflicted, or by placing a book close to his eyes and observe his efforts at reading.

No diseases are more difficult of successful treatment than those of the ear. Deeply encased within the bony enclosure, we have but limited means of investigating the changes induced by disease, and we are less familiar with diseases of the ear than any other organ. Deafness is therefore frequently assumed, but he who attempts the fraud must either be a bold or a skillful man. Those who are really deaf acquire a peculiar physiognomy and have certain gestures difficult to acquire, and are sometimes detected by a want of preparation for a sudden examination; yet the ingenuity and perseverance of some have put at fault the most skillful examination, and time and artifice have ultimately unmasked the impostor. Fodere, to whom we are indebted for most ingenious means of detecting the various frauds in disease, as well as the use of the most cruel tests, with all his observation, was frequently imposed upon. He examined a deserter alledged to be deaf. Every possible means failed, until Fodere assumed a patronizing air, assured him he should be discharged if he confessed to him privately. The man's prudence was betrayed, and to the whisper of Fodere confessed the imposture. Another instance of the intentional dropping of a piece of money, attracting the patient's attention, and thus exposing him after various tests, such as firing pistols, or platoons of musketry, without the least affecting him. A constant watch should be set upon the actions of the suspected person, and matters calculated to excite or alarm his fears should be introduced in conversation. Put your hand upon his pulse, detail some bad news or terrible disaster, or threaten punishment, and you will generally perceive its increased action. Call them suddenly by name, fire a pistol closely to the ear, rouse them from sleep, or put them under the influence of ether, and on awakening the sense of hearing may be aroused and the will not enabled to control it. A very good way to test the truth or falsehood of a case occurred in a neighboring city a few years since. A man presented himself and by signs requested alms. The physician suspecting fraud, motioned him to sit down, and placing a note in his son's hand, told him to pretend to get that changed, but in reality to find a police officer, as this man's description answered well that of a man accused of having committed murder the night before, and he would have him arrested. The boy had scarcely left before the beggar hastily departed.

To assume to be deaf and dumb requires extraordinary skill, yet our books are full of cases resisting the most rigid and persistent examination. The means used to detect the imposition of deafness may be successfully employed in such cases as firing a pistol, or communicating startling or threatening information when examining the pulse. The double infliction is seldom present unless it be congenital, as the powers of articulation are never lost unless there be some cause appreciable to medical enquiry. Indeed it is a question whether the entire absence of the tongue is a sufficient cause for muteness, as cases are recorded of persons articulating without the tongue. In these instances the muscles belonging to the tongue probably were not deficient. As a general rule it may be stated that if a person not deaf can *move* his tongue he is not dumb, as nothing short of complete paralysis can account for his being dumb. Three cases are recorded of the entire loss of tongue, yet articulation was distinct—a Portuguese girl, a boy having lost his tongue by gangrene, and an American girl—the history of each interesting and instructive. Also the history of Victor Fay, detected by Fodere, is one of the most successful and persistent cases of assumed muteness on record. He persisted two years; deceived the most scientific men in three countries, and finally was detected by mutes in a school designed for their special education. Those possessing Fodere's work will find in this case abundant food for reflection, and will be guarded in hasty prognosis.

We are frequently consulted by parents in relation to children at certain ages not talking, and our utmost skill and character are sometimes necessary to quiet apprehensions, or prevent the charlatans receiving credit for that which medical acumen and time will avert. A professor in a neighboring city lost the confidence of a family by not appreciating the fears and apparently sympathizing with them, or not sufficiently informing them, in the case of a child unable to articulate. She was five years old and had not spoken; heard very well; was sprightly and intelligent, but labored under chorea, and as a consequence the general health of the child was impaired. The professor prescribed a general routine, gave but little information as to the origin of the defection, and was superceded by a specialist, a publishing ear doctor, who carefully examined the case, observed the free motions of the tongue, prescribed a change of air, generous diet and preparations of Iron, with the certain promise of the speedy acquisition of the power of language. The case terminated favorably, and a flaming certificate brought many patients to the *skillful aurist*.

Diseases consisting of whole groups of symptoms are sometimes feigned. A disease to which all are subject and which probably destroys primarily or consecutively more lives than others, is fever, which can be most fully counterfeited. The frequency of the pulse, chattering of the teeth and hot skin, may all be induced for effect. The use of strong stimulants, as brandy, gin or cantharides, also the introduction of capsicum, garlic or cloves into the rectum will produce a glow of skin similating fever. The brown tongue of Typhoid Fever is readily produced by Ext. Liquorice or coffee browned. I saw a family thrown into the greatest distress by a favorite son having been brought home frightfully excited, with violent vomiting and dilated pupils. The head was hot and between the paroxysms of vomiting; the skin was intensely hot; pulse irregular, sometimes frequent, then slow; articulation indistinct. Cold was applied to the head and sponging to the body during the excitement, and after that stage had passed warm brandy toddy restored strength and consciousness. The boy had made his first assay on manhood that day, by trying to learn to chew tobacco and had incautiously swallowed the juice. These symptoms were indicative of a severe paroxysm of fever, violently implicating the brain and stomach. Violent exercise with friction may excite the warmth and redness of the skin, and these present with a colored tongue, all the phenomena of fever. A few hours watching, if any doubts exist, will remove the obscurity, as the symptoms in these cases rapidly subside. A profoundly intoxicated man would embarrass a physician who failed to smell the breath of the patient, and not a few are the mistakes of casual observers, and severe animadversions have been passed on opinions of the existence of disease where intoxication only was present. I remember a mistake of this kind which not only influenced public opinion detrimentally to the doctor, but did more to overthrow opinions of the whole class of the Thompsonian fraternity in Maryland. As other popular illusions it had overcome the bounds raised by law for the protection of life, and all men were allowed the privilege of practicing and charging, by repeal of laws existing from the origin of the State. A shoemaker in Washington county bought a patent and forgetting the maxim, *ne sutor*, etc., had so imposed on popular credulity as to have at one time the largest practice in three of the most populous counties. To test the doctor's skill (whose pretentions were the ridicule of all sensible men, and, unhappily, there as here they are not over numerous,) a profoundly intoxicated man was placed in bed by a party and the doctor suddenly summoned, and informed that from an upset from a sleigh the patient had been thrown on a curb stone and it was feared a fractured skull had resulted. The doctor carefully examined the case and very oracularly stated, "that the brain were not hurt, but that the patient would certainly die, and it was his wish that the body should be examined by the Calomel doctors, as one thing was plain—that a *Gut are broke.*" Unfortunately for the doctor, a few hours sound sleep restored the patient, and the jest was too good for silence. No other argument was used in reply to the skill and success of the doctor than the ominous words that a "gut are broke," and in a much shorter time than had sufficed to give him the reputation and practice he had acquired, he disposed of his fine horses and is now engaged in his legitimate business.

As coma from fever or injuries of the brain and spine, differs in some respects from that induced by alcohol, the nose affords the best diagnosis. Also from disease resulting from the use of tobacco, in which coma exists, we have the fœtor of the breath to direct our enquiries.

Agues are feigned, i. e. the chill part. If no fever follows, we may be assured, after frequent repetition, that this is assumed. A gentleman of my acquaintance had a negro boy whose services were valuable, and who had a recurrence of fits of ague for several weeks. Three or four days nursing and treatment would check the paroxysms; but a return was constant, and the physician finding the health of the boy not impaired, by their frequent recurrence, gave him Tinct. Ant. and Jalap. The boy returned at the usual period, and when about to receive the unpleasant compound, drew the handkerchief from his head and thought he was better, and as he could not get a seidlitz powder, declined taking anything, and did not return. As before observed, those who feign sickness are averse to active or unpleasant treatment. A mixture in the Eastern hospitals, called, for want of an official name, Mistura Diabolica, has performed most miraculous cures. It is composed of Assafœtida, Glauber's Salts and Aloes, and is given in very small doses, frequently repeated, so as constantly to impress the mouth with its nauseousness.

The diseases of the chest are more readily diagnosed, although formerly pneumonia was in the army, and now is, frequently assumed. It is unnecessary to put any educated physician on his guard in this disease, as his stethescope will detect the malinger; but by wounding the throat, hœmoptisis may be induced or pretended; mucous expectoration by constant hawking and coughing may also be produced; emaciation by abstinence or drinking acids, or by constantly sucking a copper coin may impair the tone of the stomach and thus induce disease. This is not uncommon in the army, and is traced to foreign importation.

The diseases of the abdominal cavity are assumed, and dyspepsia is the form. Care and watching can detect the fraud, as gastralgia, pyrosis and vomiting can be produced at will. Inflammation of the stomach is also assumed. The absence of the red papilla on the tongue and the long intermissions in vomiting are grounds of suspicion. Peritonitis and Hepatitis were much feigned in the British army in East India stations, and many discharges were granted soldiers for these diseases. After a time the urine was found colored with rhubarb; clay stools induced by muriatic acid; but the colored eye and skin, together with the listlessness and mental depression consequent upon this disease being absent, the surgeons suspected fraud, and the mixture Diabolica performed most remarkable cures, and was more successful than Mercury. Nephritis is sometimes assumed. This is not difficult in the male to detect, as the retraction of the testicle is the guide.

In the army, after severe attacks of Nephritis, the patients bring calculi alleged to have passed the urinary passage—found on analysis to be pebble, brick-bat or glass. In conclusion, as a general rule we would not be justified in treating disease assumed to be feigned differently from the real. Low diet, strict confinement to bed and nauseous medicines may be the effectual plan and will generally succeed. It is but fair to malinger ourselves and to lead the patients by questions into incongruous and incompatible answers, to detect by a fraud that which cunning and artifice had formed for our disgrace and their benefit. The impostor is ever averse to taking medicines, careful watching, visits at unusual and unexpected hours. This proceeding detected one case of five month's deception.

The absence of motive for assuming disease is not conclusive that it is real. Some from desire for sympathy of friends and others from no explicable cause than moral insanity, assume to be ill ; yet it is well at all times to endeavor to obtain the motive, as this may be a clue to the whole disease. The previous history of the patient and in the army the opinions of his comrades may serve to enlighten. In some cases, however, previous moral character will not be altogether reliable, as men of most unexceptional conduct have imposed on Army Surgeons. Searching the beds and pockets will sometimes reveal the irritating substance that produces an obstinate ulcer ; and if an accomplice is suspected, perfect isolation will reveal all you want—the cause and the cure; and a thorough chemical test or microscopic examination of foreign matter alleged to be discharged, should be made.

I have thrown these thoughts and compilations together with the hope some aid may thereby be given to a class of over-worked and neglected men now in our army, who are subjected to more imposition and mortification, more expected from them, and less remuneration, than any other human being. I mean Army Surgeons; and will have succeeded if I have aided even *one* in his responsible and heavy duties.

